# Assessing the Mobility of Lead, Copper and Cadmium in a Calcareous Soil of Port-au-Prince, Haiti ^†^

**DOI:** 10.3390/ijerph10115830

**Published:** 2013-11-04

**Authors:** Urbain Fifi, Thierry Winiarski, Evens Emmanuel

**Affiliations:** 1 Université Quisqueya—LAQUE, 218, Avenue Jean Paul II, Haut de Turgeau, P.O. Box 796, Port-au-Prince, HT 6113, Haiti; E-Mail: evens.emmanuel@gmail.com; 2 Université de Lyon—LEHNA, UMR 5023, ENTPE, Rue Maurice Audin, Vaulx-en-Velin CEDEX FR 69518, France; E-Mail: Thierry.winiarski@entpe.fr

**Keywords:** lead, copper, cadmium, models, soils, sorption

## Abstract

The presence of heavy metals in the environment constitutes a potential source of both soil and groundwater pollution. This study has focused on the reactivity of lead (Pb), copper (Cu) and Cadmium (Cd) during their transfer in a calcareous soil of Port-au-Prince (Haiti). Kinetic, monometal and competitive batch tests were carried out at pH 6.0. Two simplified models including pseudo-first-order and pseudo-second-order were used to fit the experimental data from kinetics adsorption batch tests. A good fit of these data was found with pseudo-second-order kinetic model which indicates the applicability of this model to describe the adsorption rates of these metals on the soil. Monometal batch tests indicated that both Langmuir and Freundlich models allowed a good fit for experimental data. On the basis of the maximum adsorption capacity (*q*_max_), the order affinity of Pb, Cu and Cd for the studied soil was Pb^2+^ > Cu^2+^ > Cd^2+^. Competitive sorption has proved that the competition between two or several cations on soils for the same active sites can decrease their *q*_max_. These results show that, at high metal concentrations, Cd may pose more threat in soils and groundwater of Port-au-Prince than Pb and Cu.

## 1. Introduction

Heavy metals ions in soils have been a very useful indicator of environmental quality worldwide. Heavy metal ions are the most toxic inorganic pollutants which occur in soils and can be of natural or of anthropogenic origin [1,2,3]. Lead, copper, and cadmium belong to the group of serious hazardous heavy metals and are generally considered a threat to human health and ecosystems because of their potentially high toxicity [4]. Their mobility in soils may be controlled by different chemical mechanisms such as surface complex formation, ionic exchange, precipitation, and adsorption processes. However, the most important chemical process that affects heavy metal availability is adsorption onto soil solid phases [5]. Their solubility and bioavailability may also be controlled by soils characteristics [6], such as pH, redox potential, clay minerals, soil organic matter, Fe and Mn oxides, and calcium carbonate. Therefore, metals adsorption and hence availability does not only depend on soil constituents (inorganic and organic), but also on the available metals, and their competition for soil sorption sites [5]. 

Many authors have investigated metals adsorption on different soils materials and under different experimental conditions [6,7,8,9,10,11,12,13,14,15,16]. Most trace element adsorption has been derived from studies conducted using single metal solutions [15,17]. Usually, single metal solutions have limited practical applications [18]. However, multi-metal solutions are extremely important for a better understanding of competitive sorption of metal ions. In addition, it is well-known that most heavy metal contamination in the surface environment is associated with a cocktail of contaminants rather than one metal.

Previous research at Port-au-Prince has showed an impact of groundwater quality related to the contribution of urban contaminants. For example, Pb concentrations ranging from 10 μg·L^−1^ to 90 μg·L^−1^ were measured in the drinking water of Port-au-Prince [19,20,21]. In this study, we have investigated the potential capacity of Pb, Cu and Cd to sorb on soils of Cul-de-Sac plain. Knowledge about the mobility of these heavy metals in soils of Port-au-Prince may play a key role in the designing of control strategies to achieve better groundwater protection.

## 2. Materials and Methods

### 2.1. Soil Samples and Characterization

**Figure 1:**
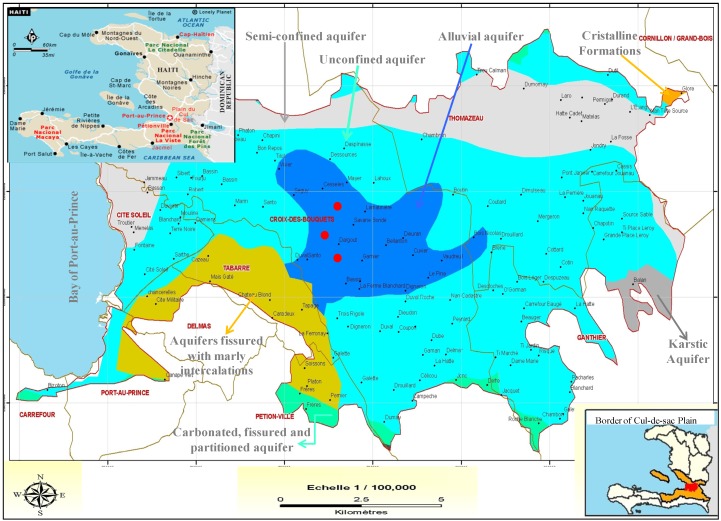
Aquifer systems of Cul-de-sac Plain, Haiti (sampling points 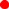).

Three approximately 3-kg soil samples from 2 m apart of the same site were collected and combined prior to the experiments from the alluvial formations of the Cul-de-Sac plain at Port-au-Prince, which is not subjected to human activities (Figure 1). 

The ≤2 mm size soil fraction was used for laboratory experiments. This grain size is most reactive [13]. In general, coarse-grained soils exhibit lower tendency for heavy metal adsorption than fine-grained soils. The fine-grained soil fraction contains soil particles with large surface reactivities and surface areas. Clay minerals, iron and manganese oxyhydroxides, humic acids, and others minerals present have enhanced adsorption properties [1]. All the samples were air-dried at room temperature, passed through a 2 mm sieve, homogenized, and stored pending measurement of physicochemical properties such as pH, organic carbon, clay, and CaCO_3_ using standard analytical methods. Soil pH was measured using a pH meter at a soil to solution ratio in both deionized water in 1:2.5 and 1 mol·L^−1^ KCl. Soil organic matter (OM) was determined by calcination at 550 °C for 2 h. The inorganic carbon was determined using the calcimeter method and carbonate concentrations were calculating using Universal Gas Law [3]. The cation exchange capacity (CEC) of the soil was determined using the Metson method [22]. Concentrations of available heavy metals in the soil samples were determined by atomic absorption spectrometry (AAS) using NF ISO 11885 guidelines. 

### 2.2. Experimental Set-up

Batch tests were carried out by equilibrating 5 g of soil with 50 mL of solutions containing different metal concentrations in 0.01 M NaNO_3_. All our experiments were performed at pH 6.0 (adjusted using dilute HNO_3_ or NaOH) in order to have a stable solution and avoid metals precipitation on hydroxides forms which can introduce uncertainty into the interpretation of results [23]. The metals cations were applied in the forms Pb(NO_3_)_2_, Cu(NO_3_)·3H_2_O and Cd(NO_3_)_2_·4H_2_O. Nitrates were used because these ions have no affinity for metals [13,24]. After equilibrium, the suspensions were filtered though a 0.45 µm membrane, and samples were carefully dispensed to 50 mL polyethylene sample cups, acidified to pH 1.5–2 using strong HNO_3_ and stored at 4 °C until the heavy metal ion measurements by AAS. 

#### 2.2.1. Adsorption Kinetics

Metal adsorption depends on the reaction kinetics and the time of contact between metal ions and soil. In this study, Kinetics batch tests were carried out at room temperature and samples were taking after 1 min, 3 min, 8 min, 15 min, 30 min, 60 min, 120 min, 360 min, 720 min, 1,440 min, 2,880 min and 4,320 min. The metal concentrations equilibrated with the soil sample were 250, 80 and 123 mg·L^−1^ of Pb, Cu and Cd respectively. The metal suspensions were prepared and analyzed by AAS.

#### 2.2.2. Monometal Adsorption

Monometal batch tests were performed over a 24 h period by shaking range concentrations of Pb (0−186 mg·L^−1^), Cu (0−57 mg·L^−1^) and Cd (0−101 mg·L^−1^) at room temperature. After equilibrium time, the suspensions were prepared for metal ions measurements by AAS. The amount of the metal ions sorbed by soil was calculated by:

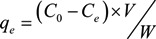
(1)
where *q_e_* is the amount of Pb^2+^, Cu^2+^ or Cd^2+^ adsorbed on the soil (mg·g^−1^), *C_e_* is the concentration of Pb^2+^, Cu^2+^ or Cd^2+^ at equilibrium (mg·L^−1^), *C*_0_ is the initial concentration of Pb^2+^, Cd^2+^ or Cu^2+^ in solution (mg·L^−1^), *V* is the solution volume (mL), and *W* is the weight of air-dried soil (g).

#### 2.2.3. Competitive Adsorption

Bi- and tri- metal batch tests were carried out by solubilizing a combination of either (Pb^2+^–Cu^2+^), (Pb^2+^–Cd^2+^), (Cu^2+^–Cd^2+^) and (Pb^2+^–Cu^2+^–Cd^2+^). These experiments were conducted with the same operating conditions as for monometal batch tests in terms of volume (50 mL), soil sample weight (5 g), heavy metals concentrations ranges, pH (6.0) and agitation time (24 h). 

### 2.3. Theory

To study the adsorption processes, simple mathematical expressions are usually applied to establish relationships between concentration of the adsorbent in the liquid phase and the solid phase at equilibrium and at constant temperature. During these experiments, adsorption processes do not always have time to reach equilibrium, but it is limited instead by reaction kinetics.

#### 2.3.1. Kinetics Models

Kinetics batch tests were performed in order to evaluate the reaction rates of Pb, Cu and Cd on the selected soil. Two simplified kinetics models including pseudo-first-order and pseudo-second-order were tested [25,26,27,28]. The pseudo-first-order equation is linearly expressed as:

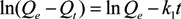
(2)
where *Q_e_* (mg·g^−1^) is the adsorption capacity at equilibrium, *Q_t_* (mg·g^−1^) is the amount of the metal adsorbed at time *t*, and *k*_1_ (min^−1^) is the rate constant of the pseudo-first-order equation. The values of *k*_1_ can be obtained from the slope of the linear plot of ln(*Q_e_* − *Q_t_*) *vs. t* at different metal concentration. The linearised form of pseudo-second-order equation [25] is expressed as:

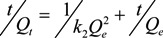
(3)
where *k*_2_ is the rate constant of pseudo-second-order kinetics. The values of *k*_2_ (g·mg·min^−1^) and *Q_e_* can be determined from the slope and intercept of the plot obtained by plotting *t/ Q_t_ vs. t* respectively.

#### 2.3.2. Isotherms Adsorption Models

Langmuir and Freundlich models were used to study monometal isotherms of Pb^2+^, Cd^2+^ and Cu^2+^ on the soil [29]. The above two models are given, respectively, as follows:

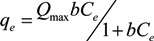
(4)

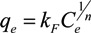
(5)
where *C_e_* (mg·L^−^^1^) and *q*_e_ (mg·g^−^^1^) are the equilibrium adsorbante concentrations in the aqueous and solid phases, respectively; *Q*_max_ is the maximum adsorption (mg·g^−^^1^) and b (L·mg^−^^1^) is the adsorption equilibrium constant; *k_F_* (L·mg^−^^1^) is the Freundlich distribution coefficient and *n* is an empirical constant (unitless).

Jain and Snoeyink (JS) [30] have proposed a modified equation of the Langmuir model Equation (4) for bi-solute adsorption systems. The extended Langmuir model takes into consideration that the presence of other metals in solution can affect the apparent affinity of the metal for the adsorption on an active site [31]. The JS modified model equations is given by:

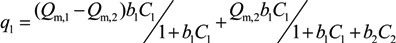
(6)

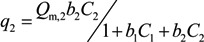
(7)
were *q*_1_ and *q*_2_ are the amount of metals 1 and 2 adsorbed per unit weight of adsorbent at equilibrium concentrations *C*_1_ and *C*_2_. The first term of the Equation (6) is the Langmuir expression for the number of molecules of solute 1 that sorb without competition on the surface area and the term is proportional to (*Q*_m,1_ − *Q*_m,2_). The second term of this equation represents the number of molecules of solute 1 sorbed on the surface area proportional to *Q*_m,2_ in competition with solute 2, and is based on the Langmuir model for competition adsorption. The number of molecules of solute 2 sorbed on the adsorbent surface is proportional to *Q*_m,2_ in competition with solute 1, can be calculated from Equation (7). The JS model was used in this study to assess the bi-metal competitive adsorption of Pb, Cu and Cd on the studied soil.

Experimental data from tri-metal batch tests was modeled using Langmuir extended model, as follows:

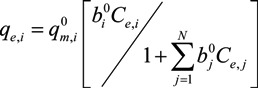
(8)
where 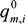, 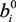
and 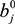 are Langmuir extended parameters obtained from Equation (4) in monometal batch tests and *C_e,i_* and *C_e,j_* are respectively the concentrations of metals *i* and *j* from tri-metal batch testsafter equilibrium.

## 3. Results and Discussion

### 3.1. Soil Characteristics

Table 1 shows the physicochemical characteristics of the studied soil. The results confirmed that the soil have an alkaline pH value (8.26) due the presence of the free CaCO_3_ (343 g·kg^−1^). The CaCO_3_ value is consistent with a watershed rich in carbonate formations. Data indicate abundant organic matter (OM) (58 g·kg^−1^) and Cation Exchange Capacity (CEC) of the soil sample. The CEC can be estimated by the clay content and organic matter. Therefore, soils with very little OM have a low CEC, but heavy clay soils with high levels of OM would have a much greater capacity to sorb cations. Soil samples had Pb and Cd concentrations below detection limits. These values have justified the choice of soil sample area. Cu concentrations (61.4 mg·kg^−1^) are probably related to natural concentrations. 

**Table 1:** Physicochemical characteristics of soil from Cul-de-Sac plain*.*

Parameters	Concentration	Standards and analysis methods
pH-H_2_O	8.26	AFNOR X31-104
pH-KCl	7.46	AFNOR X31-104
CaCO_3_ (g·kg^−1^)	343.00	AFNOR X31-105
Organic carbon (g·kg^−1^)	100.00	AFNOR X31-106
Organic matter (g·kg^−1^)	57.85	Calcination at 550 °C
Clay (g·kg^−1^)	17.00	AFNOR X31-107
CEC (meq·kg^−1^)	135.00	Metson Method AFNOR X31-130
Surface area (m^2^·g^−1^)	9.48	B.E.T Method
Total Ca (g·kg^−1^)	9.67	AFNOR X31-108
Total Mg (g·kg^−1^)	0.45	AFNOR X31-108
Total K (g·kg^−1^)	0.051	AFNOR X31-108
Total Cr (mg·kg^−1^)	17.40	NF ISO 11885
Total Cu (mg·kg^−1^)	61.40	NF ISO 11885
Total Ni (mg·kg^−1^)	24.10	NF ISO 11885
Total Zn (mg·kg^−1^)	28.10	NF ISO 11885
Total Cd (mg·kg^−1^)	Ud *	NF ISO 11885
Total Pb (mg·kg^−1^)	Ud	NF ISO 11885
Total Hg (mg·kg^−1^)	Ud	NF ISO 11885
Total Se (mg·kg^−1^)	Ud	NF ISO 11885

* Undetected.

### 3.2. Kinetics

The adsorption rates of the three metals have been evaluated using Equations (2) and (3). The obtained parameters for pseudo-first and second order are given in Table 2. The low values of correlation coefficients indicate that the pseudo-first order model is inappropriate to describe the adsorption rates processes. 

**Table 2:** Constants and correlation coefficients obtained by pseudo-first-order andpseudo-second-order kinetics models.

Metal ions	Pseudo-first order		Pseudo-second order		
	*K*_1_ (min^−1^)	*R*_1_^2^	*Q*_e_ (mg·g^−1^)	*K*_2_ (g·mg^−1^·min)	*R*_2_^2^
Pb^2+^	0.00139	0.66	2.50	0.25	1.00
Cu^2+^	0.00147	0.68	0.79	0.77	1.00
Cd^2+^	0.00010	0.83	1.24	0.01	0.99
Pb^2+^ (Pb^2+^–Cu^2+^–Cd^2+^)	0.00047	0.48	2.61	0.075	1.00
Cu^2+^ (Cu^2+^–Pb^2+^–Cd^2+^)	0.0012	0.71	0.86	0.055	0.99
Cd^2+^ (Cd^2+^–Pb^2+^–Cu^2+^)	0.00073	0.91	1.58	0.002	0.94

**Figure 2:**
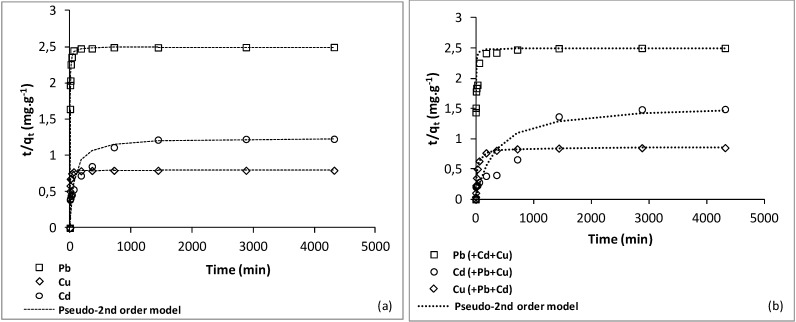
Pseudo-second order kinetics plots of Pb^2+^, Cu^2+^ and Cd^2+^ in the soil: (**a**) Monometal batch tests; (**b**) Tri-metal batch tests.

The pseudo-second order kinetic plots (*t/q*_t_ *vs. t*) appeared to give a better understanding of the interactions (Figure 2). However, the good fitting (*R*^2^ = 1.0) of the experimental data for Pb^2+^ and Cu^2+^ ions with pseudo-second-order model indicates the applicability of this model to predict adsorption rates for each metal on the soil. It was denoted that a pseudo-second-order approach can sometimes provide a better description of the adsorption kinetics [32,33]. 

Results from monometal batch tests have showed that Pb displayed the fastest adsorption rates comparatively to Cu and Cd (Figure 2a). Therefore, at about 180 min, the maximum adsorption capacity of Pb and Cu were obtained whereas Cd had continued to sorb over 4,320 min. Results from tri-metal batch tests have proved that a decrease of adsorption rates for the three metals (Figure 2b). These results have showed that the maximum adsorption capacity for Pb, Cu and Cd was obtained respectively at 2,880, 4,320 and over 4,320 min. These results showed that when two or more metal ions are together in soils, their adsorption rates is decreased each other. Therefore, their mobility in soils can be limited by competition for the adsorption sites and they don’t represent a potential risk at short-term for groundwater of Port-au-Prince. 

### 3.3. Monometal Adsorption

**Figure 3:**
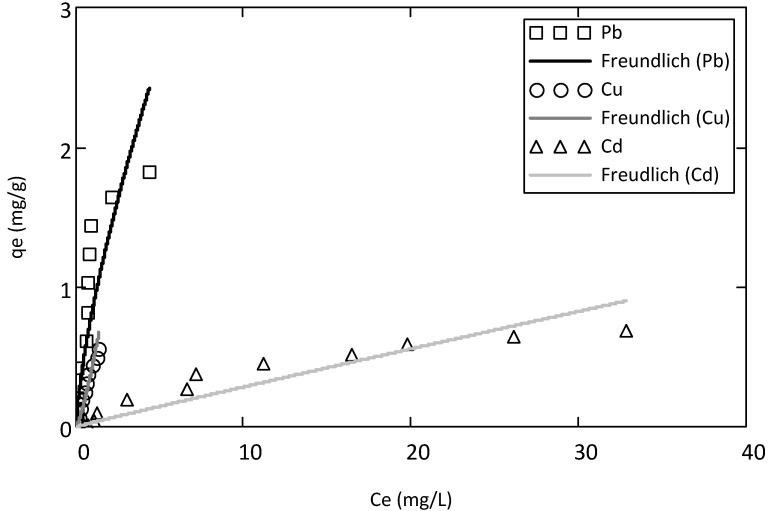
Freundlich adsorption isotherm for Pb^2+^ Cu^2+^ and Cd^2+^ on the studied soil at pH 6.

**Figure 4:**
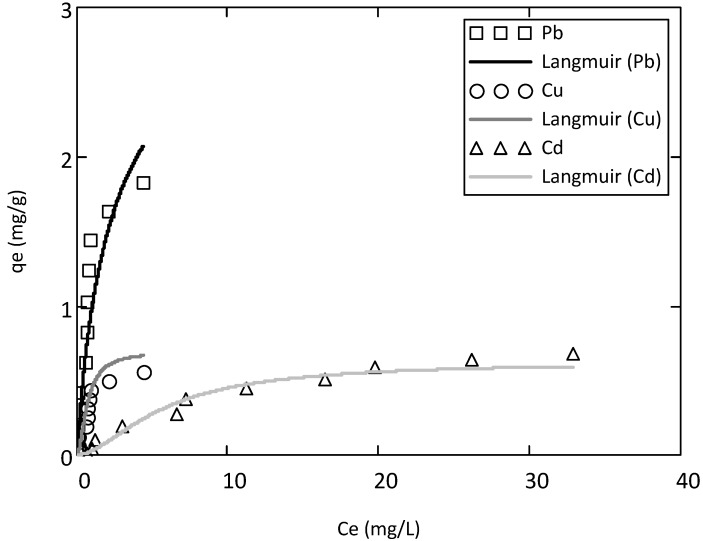
Langmuir adsorption model for Pb^2+^, Cu^2+^ and Cd^2+^ on the studied soil at pH 6.

The adsorption isotherms of Langmuir and Freundlich for Pb, Cu and Cd ions at pH 6.0 are illustrated in Figure 3 and Figure 4, respectively. These isotherms represent the adsorption behavior of these metals on the soil as a function of increasing aqueous metal ion concentration after equilibrium. The results indicated that the adsorption data of the three metals were well correlated with Langmuir and Freundlich models. The Freundlich equation habitually provides a good description of adsorption onto heterogeneous solid surfaces [34,35]. However, the adsorption of Pb^2+^ data gave a good satisfactory fit with both Langmuir (*R*_L_^2^ = 0.91) and Freundlich (*R*_F_^2 ^= 0.91). The *q*_max_, *b*, *R*_L_^2^ (correlation coefficient for Langmuir isotherm); *K*_F_, n and *R*_F_^2^ (correlation coefficient for Freundlich isotherm) are given in Table 3.

Freundlich parameters (*K*_F_ and *n*) indicate whether the nature of adsorption is either favorable or unfavorable [36]. The values of *n* are less than 1 indicate a favorable adsorption mechanisms and formation of relatively stronger bonds between the adsorbents [37]. In Table 3, the low values of *n* (*n* <1) for Pb*^2+^* and Cd^2+^ indicate that adsorption intensity is favorable at high range of concentrations studied, while for Cu2+ (n > 1) means that adsorption intensity is unfavorable at high concentrations but much less at lower concentrations. Some studies on other sites area have supported this conclusion [34,36,38,39]. 

The *q*_max_, from the Langmuir equation, may be a useful parameter for comparing the potential capacity of the soil. Among all the three metals, Pb showed the highest value of adsorption maximum (*q*_max_). On the basis on the *q*_max_ value, the order selectivity of these metals for the soil is Pb^2+^ > Cu^2+ ^> Cd^2+^. The selectivity order can be influenced by the valency and the ionic size of the heavy metals once hydrated [40,41]. Then, smaller ions with the same valency, such as Cd compared with Pb, have higher charge densities and attract more water molecules, resulting in a larger hydrated radius. Metals with higher hydrated radius exert weaker Columbic forces of attraction [42]. Therefore, Cd (0.23 nm radius) is expected to be mobile that Pb (0.187 nm radius) because of its larger hydrated radius. In order case, the higher affinity of the soil for Pb may probably due to the existence of a greater number of active sites (mostly organic matter) with high specificity for Pb, so when it is present these sites would not be occupied by others cations. According to these results, Cd may pose more threat to soils and groundwater of Port-au-Prince than Pb and Cu. These results strongly suggest why Berti and Jacobs [43] found that soil loading of Cd, Ni, and Zn appeared to be of greater environmental concern than Cr, Cu, and Pb and that the first group could accumulate in the tissue of plants grown on sludge-treated plots [41].

### 3.4. Competitive Adsorption

Competitive adsorption studies were useful to assess the degree of interference posed common metal ions in the soil. The parameters of the JS and Langmuir extended models used in this study are summarized in Table 3. It was observed that Pb was always favorably sorbed on the soil over Cu and Cd in all the experiments. The experimental data from Pb and Cu bi-metal batch tests were better fitted than Cd with the JS model. The geochemical behavior of the three metals was evaluated following to their maximum adsorption capacity in the soil. These results indicated that competitive between the three metals have been reduced the adsorption capacity in the soil. Qin *et al.* [4] suggested that when two or more metal ions are present together, they may increase, decrease or not change the metal-ion adsorption capacity of the adsorbent. The competitive of Cd and Pb in acid soils was studied by Serrano *et al.* [15] and they noted that the co-existence of Pb and Cd reduces their tendency to be sorbed on the soil solid phases, thereby affecting the adsorption capacity of Cd to a greater extent than Pb. The same phenomenon was observed by Morera *et al.* [44] using competitive adsorption isotherms to evaluate the mobility of Cd, Cu, Ni, Pb and Zn in four soils differing in their physicochemical properties.

**Table 3:** Isotherm adsorption parameters for Pb, Cu and Cd in monometal and bi-solutes systems on the soil (*q*_max_L; *q*_max_JS: mg·g^−1^; *b*_L_, *b*_JS_, *K*_F_: L·mg^−1^).

Metals	Adsorption batch tests	Langmuir parameters			Freundlich parameters		
	Monometal	*q*_max_L	*b*_L_	*R*_L_^2^	1/n	*K*_F_	*R*_F_^2^
Pb	Cd^2+^	3.64	0.37	0.91	1.41	0.85	0.91
Cu	Cu^2+^	0.70	1.81	0.91	0.78	0.45	0.92
Cd	Cd^2+^	0.63	0.05	0.90	1.01	0.03	0.89
	**Bi-metal**	**Jain and Snoeyink parameters**					
		***q*_max_JS**	***b*_JS_**	***R*_JS_^2^**	**Δ*q*_JS_ (%)**	***r*_JS_**	
Pb	(Pb^2+^–Cd^2+^)	3.09	0.36	0.99	15.11	0.85	
	(Pb^2+^–Cu^2+^)	2.95	1.40	0.97	18.95	0.81	
Cu	(Cu^2+^–Cd^2+^)	0.59	2.07	0.98	15.71	0.84	
	(Cu^2+^–Pb^2+^)	0.45	1.63	0.94	35.71	0.64	
Cd	(Cd^2+^–Pb^2+^)	0.46	0.09	0.87	26.98	0.73	
	(Cd^2+^–Cu^2+^)	0.10	0.44	0.75	84.13	0.16	
	**Tri-metal**	**Extended Langmuir parameters**					
		***q*_max_LE**	***b*_LE_**	***R*_LE_^2^**	**Δ*q*_LE_ (%)**	***r*_LE_**	
Pb	(Pb^2+^–Cu^2+^–Cd^2+^)	0.77	1.56	0.95	78.86	0.21	
Cu	(Cu^2+^–Pb^2+^–Cd^2+^)	0.43	1.79	0.98	38.57	0.61	
Cd	(Cd^2+^–Pb^2+^–Cu^2+^)	0.10	0.85	0.91	84.13	0.16	

Mohan and Singh [45] have investigated the mutual effects of metals ions on their adsorption in multi-solute system by measuring the adsorption capacity ratio of one metal in multi-solute, 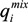 and 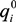 the single-solute system, , following this equation:

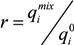
(9)
where 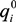 and 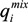 are the maximum amount sorbed according to monometal or multi-metal batch tests, respectively and *r* is the adsorption capacity ratio. If *r* > 1, metal *i* enhanced the adsorption of the others ions. If *r* = 1, metals had no effects on each other. If *r* < 1, metal *i* completed for with other metals for the adsorption sites of adsorbents. As showed in Table 3, all the values of *r* are lower than 1 which indicates the mutual competitive effect of each metal in all the experiments. The *r* values obtained from tri-metal batch tests are lower than those from bi-metal adsorption systems. These results indicated that the competitive adsorption processes depend on the quantity of metals ions from solid and liquid phases.

The rate of adsorption reduction (Δ*q*) can be calculated following the Equation (10). This rate is the ratio of the difference between non-competitive and competitive adsorption observed at equilibrium:

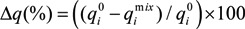
(10)


According the Δ*q*, Pb, Cu and Cd ions had different competitive effect. For Pb, the adsorption capacity was reduced by 15.11%, 18.95% and 78.86% respectively in (Pb^2+^–Cd^2+^), (Pb^2+^–Cu^2+^) and (Pb^2+^−Cu^2+^−Cd^2+^) systems. Similarly, the rate of adsorption equilibrium reduction of Cu, comparing to its adsorption in monometal, decreased respectively by 15.71%, 35.71% and 38.57% in (Cu^2+^–Cd^2+^), (Cu^2+^–Pb^2+^) and (Pb^2+^–Cu^2+^–Cd^2+^) systems. Finally, for Cd, its adsorption capacity was reduced by 26.98% in (Cu^2+^–Pb^2+^), by 84.13% in both (Cd^2+^–Cu^2+^) and (Pb^2+^–Cu^2+^–Cd^2+^) systems. Therefore, the similarity between the Δ*q* of Cd in (Cd^2+^–Cu^2+^) and (Pb^2+^–Cu^2+^–Cd^2+^) systems may indicate that Cu can suppress Cd adsorption greater than Pb. According to the different rates of adsorption equilibrium reduction effect, the affinity sequence of the three metals for the soil in tri-metal adsorption systems is Cu^2+^ > Pb^2+^ > Cd^2+^. That means, when the three metals are in competition for the same sorption sites, Cu could displace Pb and Pb could displace Cd. Indeed, the affinity order found from monometal adsorption batch tests, Pb^2+^ > Cu^2+^ > Cd^2+^, remained the same in competitive batch tests. In spite of the maximum capacity of Pb decreased related to competitive adsorption, it was mostly adsorbed on the soils over Cu and Cd.

## 4. Conclusions

This study has shown in general that the soil of Port-au-Prince has a high capacity to sorb metal ions. Results from kinetics batch tests have shown the applicability of a pseudo-second order model to describe the adsorption rates of each metal on the soil. The ranked affinity of the selected metals for the soil was Pb^2+^ > Cu^2+^ > Cd^2+^ according to the maximum adsorption capacity obtained by the Langmuir model. Results from multimetal batch tests indicated that competition between heavy metals for sorption sites can reduce their maximum adsorption capacity on the soil. On the basis of results from this study, Cd may pose more threat to soils and groundwater of Port-au-Prince than Pb and Cu. In short, regular groundwater samples and analysis may be carried out to assess changes in groundwater quality. It’s necessary also to complete this study by coupling chemistry with a transport model for a better understanding of heavy metals transfer mechanisms to groundwater of Port-au-Prince.
